# Medial Thigh Lift with Tumescent Local Anesthesia: Advancing Outpatient Body Contouring

**DOI:** 10.3390/jcm14165630

**Published:** 2025-08-08

**Authors:** Federico Ziani, Edoardo Filigheddu, Giovanni Arrica, Sofia De Riso, Gianluca Marcaccini, Roberto Cuomo, Claudia Trignano, Corrado Rubino, Emilio Trignano

**Affiliations:** 1 Department of Medicine, Surgery and Pharmacy, University of Sassari, 07100 Sassari, Italy; zianifederico@gmail.com (F.Z.); edofili@gmail.com (E.F.); giovanni.arrica@aouss.it (G.A.); s.deriso1@studenti.uniss.it (S.D.R.); corubino@uniss.it (C.R.); etrignano@uniss.it (E.T.); 2 Plastic Surgery Unit, University Hospital Trust of Sassari, 07100 Sassari, Italy; 3 Plastic and Reconstructive Surgery, Department of Medicine, Surgery and Neuroscience, University of Siena, 53100 Siena, Italy; robertocuomo@outlook.com; 4 Department of Biomedical Sciences, University of Sassari, 07100 Sassari, Italy

**Keywords:** tumescent local anesthesia, medial thigh lift, body contouring, outpatient surgery, patient safety, esthetic plastic surgery

## Abstract

**Background**: Demand for anesthesia-sparing body contouring techniques is rising. This study assessed the feasibility, safety, and outcomes of medial thigh lift performed exclusively under tumescent local anesthesia (TLA) in an outpatient setting. **Methods**: A retrospective review was conducted that included 43 female patients (mean age of 41.6 years; BMI of 27.6 kg/m^2^) treated from November 2019 to June 2023. All procedures used pure TLA without sedation; a horizontal excision alone or combined with liposuction was chosen according to preoperative evaluation. The end-points were operative time, intra-operative pain (four-point scale), complications, and 12-month patient satisfaction. **Results**: Surgery was completed under TLA in every case, with no conversion to general anesthesia. The median operative time was 30 min for excision-only procedures and 50 min when combined with liposuction. Intra-operative comfort was rated “excellent” (86.0%) or “good” (14.0%); no opioids were required postoperatively. The overall complication rate was 23.2% (10/43), limited to minor wound dehiscence (9.3%), dog-ear deformity (7.0%), and scar displacement/hypertrophy (7.0%). No seroma, hematoma, infection, thromboembolic events, sensory deficits, or hospital readmissions occurred. All patients were discharged after 4 h and resumed ambulation within 24 h. At 12 months, 97.7% reported being “very satisfied” or “satisfied.” **Conclusions**: Medial thigh lift under pure TLA provides reliable anesthesia and hemostasis, minimizes perioperative morbidity, and enables same-day discharge, with high patient satisfaction. The low incidence of only minor complications supports TLA as a safe, effective, and resource-efficient alternative to general anesthesia for selected patients with mild-to-moderate thigh laxity. Further comparative and long-term studies are warranted.

## 1. Introduction

Body contouring procedures have gained increasing prominence in esthetic surgery. This growth is attributed to increased demand for minimally invasive techniques that emphasize safety, efficient recovery, and enhanced patient comfort. Among these procedures, the medial thigh lift represents a crucial intervention for patients seeking to improve thigh contour and firmness following massive weight loss, aging, or lipodystrophic changes. Traditionally performed under general anesthesia, recent developments in tumescent local anesthesia (TLA) have begun to redefine the anesthetic approach to body contouring procedures. TLA offers significant advantages such as improved safety, reduced systemic risk, and expedited recovery [1].

Klein originally popularized the tumescent technique in the context of liposuction [2]. The technique involves subcutaneous infiltration of large volumes of a dilute solution containing lidocaine, epinephrine, and saline. This infiltration creates hydrodissection and vasoconstriction [3] while providing profound regional anesthesia without the need for sedation or general anesthesia [4,5]. Since its introduction, the scope of TLA has expanded beyond liposuction [6,7]. The technique has been safely utilized in abdominoplasty [8], gluteal augmentation [9], and arm contouring [10]. Studies demonstrate reductions in intraoperative blood loss, postoperative pain, and anesthetic-related complications [11,12,13].

Despite these advances, medial thigh lift procedures under TLA remain underrepresented in the literature. The thigh region poses unique challenges [14] due to its rich lymphatic networks, mobility-induced tension, and higher infection risk. However, the potential for a safe and reproducible technique under local anesthesia represents an important area of investigation. Recent studies suggest that tumescent infiltration provides effective analgesia and hemostasis in thigh liposuction procedures, as demonstrated by Wollina et al. [15]. However, their surgical protocol employed general anesthesia for the lifting component.

Contemporary healthcare delivery models emphasize the development of efficient protocols that maintain safety standards. TLA aligns with this approach by eliminating the need for general anesthesia, reducing operating room time, and facilitating ambulatory surgery models [16]. In high-risk patients with comorbidities, this technique may represent a significant advancement in surgical eligibility and accessibility [17].

This study aims to address the existing knowledge gap by describing and evaluating a comprehensive technique for performing medial thigh lift surgery exclusively under tumescent local anesthesia. Through a detailed operative protocol, perioperative management, and outcome analysis, we demonstrate that medial thigh lift under TLA can be a safe, effective, and patient-centered alternative to traditional anesthetic approaches. By challenging the conventional approach that deep-plane body contouring procedures require general anesthesia, this work proposes a novel model for advancing body contouring surgery toward anesthesia-sparing, outpatient-based practice.

## 2. Materials and Methods

### 2.1. Study Design and Setting

Between November 2019 and June 2023, 43 female patients underwent medial thigh lift procedures in an accredited outpatient surgical facility. All procedures were performed by a surgical team comprising a board-certified plastic surgeon, an assistant surgeon, a certified operating room nurse, and a board-certified anesthesiologist.

### 2.2. Preoperative Evaluation and Consent

All patients received detailed information regarding the nature of the medial thigh lift procedure, including its indications, anticipated benefits, and potential complications. The potential complications discussed included hematoma formation, seroma development, wound dehiscence, and dystrophic scarring. Written informed consent was obtained from each patient. The preoperative assessments included routine hematological evaluation and cardiac assessment to ensure surgical fitness. In patients receiving medications that interfere with the coagulation cascade (e.g., antiplatelet agents or anticoagulants), therapy was either temporarily discontinued or converted to safer alternatives according to international perioperative management guidelines. In patients requiring perioperative adjustment, antiplatelet or anticoagulant therapy was generally discontinued 5 to 7 days before surgery and resumed within 24 to 48 h postoperatively, depending on bleeding risk and clinical stability, in consultation with the prescribing physician.

### 2.3. Patient Selection Criteria

Inclusion Criteria:

- Presence of significant medial thigh skin laxity, either age-related or following massive weight loss;
- Stable body weight for at least 6 months prior to surgery;
- Body mass index (BMI) ≤ 35 kg/m^2^;
- American Society of Anesthesiologists (ASA) physical status I or II;
- Absence of active infections, uncontrolled comorbidities, or psychiatric disorders.

Exclusion Criteria:

- ASA class ≥ III;
- Active smoking or smoking cessation < 4 weeks;
- BMI > 35 kg/m^2^;
- Active dermatological conditions in the surgical area;
- History of lower limb lymphedema;
- Non-compliance with postoperative protocols or unrealistic expectations.

### 2.4. Patient Subgroup Classification

Of the 43 patients, 30 underwent combined medial thigh lift with concurrent liposuction, while 13 received medial thigh lift alone. The decision to combine liposuction was made during preoperative assessment based on the presence of persistent subcutaneous adiposity with limited potential for cutaneous retraction. Patients with predominantly redundant skin and minimal adipose volume were selected for excisional lift only.

### 2.5. Anesthetic Protocol

#### Tumescent Local Anesthesia Preparation

All procedures were performed under pure tumescent local anesthesia (TLA), without general anesthesia or intravenous sedation. Patients remained fully awake and responsive throughout the operation. This approach enabled a completely outpatient-based surgical protocol without the need for anesthetic monitoring beyond standard vital signs [18]. The tumescent solution was prepared by combining 25 mL of 2% lidocaine (500 mg), 8 mmol of sodium bicarbonate (672 mg), and 1 mL of epinephrine (1:1,000,000) in 1000 mL of 0.9% normal saline.

### 2.6. Infiltration Technique

The solution was infiltrated into the superficial subcutaneous plane of the medial thigh using a blunt-tip infiltration cannula, ensuring even distribution within the adipose tissue. The volume infiltrated varied according to skin laxity, tissue thickness, and patient body weight, typically ranging from 150 to 300 mL per thigh. A waiting period of 20 to 30 min followed infiltration to allow the full pharmacodynamic effect of lidocaine and epinephrine [5]. This ensured complete analgesia of the subcutaneous plane and maximal vasoconstriction. Prior to incision, supplemental infiltration of 1% lidocaine with epinephrine 1:100,000 was performed along the skin markings for enhanced patient comfort.

### 2.7. Dosage and Safety Considerations

The maximum lidocaine dose was calculated for each patient based on body weight. Although the classical upper limit is 7 mg/kg when combined with epinephrine, the safety margin is significantly higher in tumescent anesthesia. The recent literature supports the notion that safe use takes place at doses between 28 and 55 mg/kg due to delayed systemic absorption in fatty tissue [5,19,20,21]. These limits were strictly observed throughout the procedure to ensure patient safety. No patients required conversion to sedation or general anesthesia. Throughout the operation, patients were monitored by a board-certified anesthesiologist, with continuous assessment of heart rate, blood pressure, and comfort level.

### 2.8. Surgical Technique

#### 2.8.1. Preoperative Marking

Preoperative markings were performed with the patient in an upright position. The amount of skin requiring removal was estimated, and adipose deposits requiring liposuction were marked. The upper incision line was drawn in the inguinal groove, with the anterior end extending to the femoral triangle and the posterior end reaching the middle of the gluteal groove. The inferior line was then marked based on the amount of skin to be removed as calculated by the pinch test, creating an ellipse typically measuring 4 to 6 cm in width. The patient was then positioned on the operating table in a gynecological position. This marking protocol was based on established principles described by Lockwood, with adaptations reflecting clinical experience and modifications consistent with posteriorly shifted designs as used in profunda artery perforator (PAP) flap harvest [22,23].

#### 2.8.2. Operative Procedure

With the patient positioned in a semi-frog-leg supine position, the tumescent solution was infiltrated into the medial thigh as previously described. A 20 to 30 min delay ensured complete analgesia [24]. In the medial-thigh-lift-only group, incisions were made with a No. 15 blade directly along the preoperative markings. In the combined group, liposuction was performed first through small stab incisions using a 2 mm multiport cannula. Tunnels were created without suction, followed by manual liposuction using a syringe pump. In selected cases, a 3 mm flat cannula was used for additional contouring. Bimanual palpation was employed to ensure symmetry and avoid over-resection.

Following liposuction, the marked crescent of skin and fat was excised. Deep dissection proceeded to identify Colles’ fascia, which was anchored to the dermal layer of both wound margins using 2-0 Vicryl sutures, in accordance with Lockwood’s principles. No undermining, de-epithelialization, or drain placement was performed.

Closure was performed using barbed monofilament running in a subcuticular suture (3-0 Monocryl). A light compressive dressing and a standardized elastic compression garment were applied.

#### 2.8.3. Postoperative Care

Following surgery, all patients were discharged after 4 h of observation in the outpatient facility. A compression garment (elastic shorts or specialized thigh binder) was applied immediately postoperatively and worn continuously for 4 weeks, except during hygiene tasks. Patients received oral antibiotic prophylaxis for 5 days (either amoxicillin/clavulanic acid 875 mg/125 mg twice daily or ciprofloxacin 500 mg twice daily, depending on the patient’s allergy status). Standard postoperative follow-up was scheduled at Day 1; Weeks 1–2; Months 1, 3, and 6; and 1 year. No surgical drains were used, and patients were encouraged to resume light ambulation within 24 h.

### 2.9. Outcome Assessment Protocol

A standardized protocol for outcome assessment was employed. All patients were evaluated intraoperatively, in the immediate postoperative period (within 4 h), and at 24 h postoperatively.

Pain intensity and satisfaction with the anesthetic technique were assessed using a 4-point verbal rating scale, ranging from “unsatisfactory” to “excellent,” during structured interviews.

Additionally, all patients were classified according to the Pittsburgh Rating Scale for Aesthetic Body Contouring. This allowed preoperative stratification of skin excess and contour deformities to correlate outcomes with baseline body status.

## 3. Results

### 3.1. Patient Demographics and Baseline Characteristics

A total of 43 patients underwent medial thigh lift procedures between November 2019 and June 2023. The mean age was 41.6 years (range 28–61 years), and the mean BMI was 27.6 kg/m^2^ (range 23.0–33.0 kg/m^2^). Thirty patients (69.8%) underwent a combined procedure consisting of thigh lift with liposuction, while 13 patients (30.2%) received excision-only medial thigh lift. According to the Pittsburgh Rating Scale, most patients presented with moderate skin laxity (Grade 2, n = 36, 83.7%), while six patients (14.0%) showed mild deformity (Grade 1), and one patient (2.3%) had severe excess tissue (Grade 3). Details of the individual patient characteristics and outcomes are summarized in Table 1.

### 3.2. Procedural Efficacy and Operative Outcomes

All procedures were completed under tumescent local anesthesia (TLA) without sedation or general anesthesia. No intraoperative conversion to alternative anesthetic techniques was required. The mean operative time was 30 min for excision-only thigh lift and 50 min for combined liposuction and excision procedures.

### 3.3. Intraoperative Anesthetic Tolerance

Intraoperative pain was assessed using a standardized four-point verbal rating scale. Thirty-seven patients (86.0%) rated their intraoperative experience as “excellent”, while six patients (14.0%) described it as “good”. No patients reported their pain management as “poor” or “unsatisfactory”.

### 3.4. Postoperative Pain Management

On the first postoperative day, all patients (n = 43, 100%) reported localized discomfort at the surgical site. Pain intensity was mild to moderate and was controlled with standard oral analgesics, such as paracetamol. No patients required escalation to opioid medications.

### 3.5. Complications and Safety Profile

**Figure 1:**
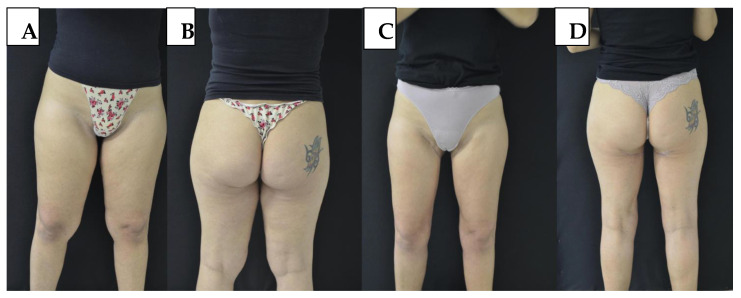
(**A**,**B**) Preoperative views of a patient presenting with localized medial thigh adiposity. (**C**,**D**) Six-month postoperative views following horizontal medial thigh lift under tumescent local anesthesia. The procedure involved skin excision only, without associated liposuction.

The overall complication rate was 23.2% (n = 10/43), with most events being minor and self-limiting. The most common complication was partial wound dehiscence, occurring in four cases (9.3%), all managed conservatively without surgical intervention. One patient (2.3%) experienced scar displacement, and 2 patients (4.7%) developed hypertrophic scars, which were treated using silicone gel sheets and corticosteroid infiltration. Three patients (7.0%) presented with dog-ear deformities at the wound edge, which were revised under local anesthesia in an outpatient setting. No cases of seroma, hematoma, infection, thromboembolic events, or long-term sensory disturbances were observed, and no patients required hospital admission postoperatively. Figure 1 illustrates pre- and postoperative views of a patient undergoing horizontal medial thigh lift without liposuction. When stratified by procedure type, the excision-only group (n = 13) experienced two complications (15.4%), while the combined excision + liposuction group (n = 30) showed eight complications (26.7%). Although not statistically significant due to the small sample size, this difference suggests a potentially higher rate of minor complications in combined procedures, likely reflecting the added tissue manipulation and liposuction volume.

## 4. Discussion

### 4.1. Tumescent Local Anesthesia in Body Contouring

Since its original development by Klein for liposuction procedures [2,4], tumescent local anesthesia (TLA) has progressively gained acceptance across a broad range of esthetic surgeries, including breast augmentation [25,26], gluteal enhancement [27], abdominoplasty [8], and arm contouring [28]. Its increasing adoption is attributed to its favorable safety profile, ability to eliminate general anesthesia, and improved pain control.

Our study presents a novel method of medial thigh lift surgery performed exclusively under TLA, without sedation, general anesthesia, or drains. This contrasts with most published studies [29,30], where medial thigh lift is traditionally performed under general anesthesia, often involving extensive undermining, vertical excision patterns, or a combined approach, depending on the grade of deformity [31].

### 4.2. Comparison with Existing Literature

The technique we describe uses a purely horizontal excision along the inguinocrural crease, a concept originally systematized by Lockwood [22] through fascial anchoring to Colles’ fascia to minimize scar migration and improve contour stability. This approach was chosen based on the limited skin redundancy and adequate tissue elasticity of our patient cohort. All patients included in this study fell within grades 1 to 2 of the Pittsburgh Rating Scale, making them suitable candidates for a horizontal-only excisional pattern.

The recent systematic review by Albanese et al. [32] evaluated 1113 patients undergoing medial thigh lift across 19 studies. Their findings highlighted a reduced overall complication rate when liposuction was integrated into the procedure (36.75% vs. 70.68%, *p* < 0.001), especially in vertical and T-shaped approaches. However, the horizontal technique alone showed one of the lowest complication rates across all groups analyzed. This supports our choice of using the horizontal approach alone in selected cases. Albanese et al. also reported that the horizontal lift technique, especially when combined with fascial fixation to Colles’ fascia, yields stable results while reducing the risk of scar migration and postoperative dehiscence, which aligns with our results.

**Figure 2:**
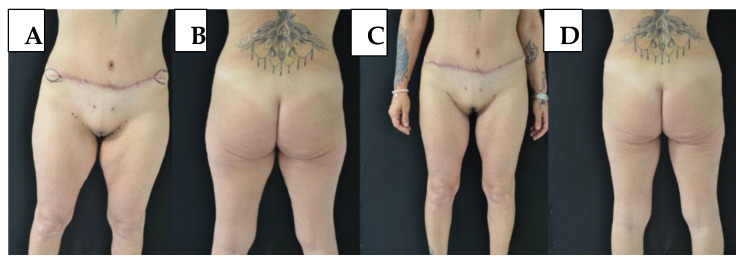
(**A**,**B**) Preoperative views. (**C**,**D**) Twelve-month postoperative views following medial thigh lift and liposuction under tumescent local anesthesia, showing improved contour and scar positioning. Note the stable scar along the inguinocrural crease and the preserved appearance of the lower abdomen.

Di Pietro et al. [33] proposed the Liposuction-Assisted Medial Thigh Lift (LAMeT) technique for post-bariatric patients. Their method combines vertical excision with extensive liposuction to preserve lymphatic and vascular structures, reduce dead space, and avoid the use of drains. While their patient population presented with more advanced deformities, the technique emphasizes tissue mobilization via liposuction rather than undermining. Although our approach did not systematically include liposuction, it was selectively employed in some cases to assist contouring while maintaining the benefits of limited dissection under tumescent local anesthesia. Figure 2 depicts the esthetic outcome and scar positioning in a patient treated with combined excision and liposuction under TLA.

### 4.3. Technical Advantages of TLA

**Figure 3:**
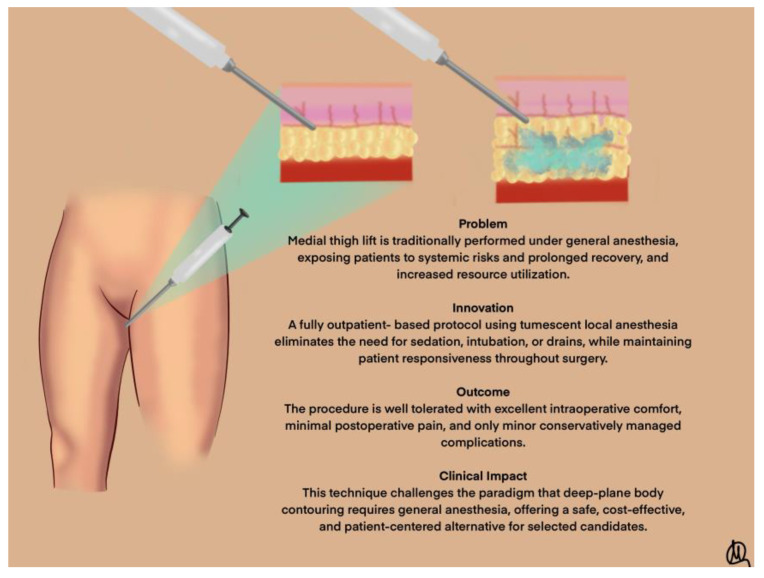
Schematic representation of procedure’s key elements.

Our data demonstrate that medial thigh lift under TLA is feasible and well tolerated, with no intraoperative conversion to general anesthesia. The anesthetic and vasoconstrictive properties of the tumescent solution allowed for safe dissection and hemostasis even in anatomically challenging regions such as the femoral triangle and perineal crease. The absence of systemic sedation or intubation reduced cardiopulmonary risks, while same-day discharge was achieved in all patients. A schematic representation of the procedure’s key elements is shown in Figure 3.

### 4.4. Complication Management and Safety Profile

The complication rate in our cohort (23.2%) is lower than that reported in the broader literature. In a comprehensive review by Sisti et al. [29], the overall complication rate for medial thigh lift reached 42.7%, with wound dehiscence and seroma being the most frequent events. In our series, all complications were minor and managed conservatively, with no cases of hematoma, seroma, infection, thromboembolism, or sensory loss. We attribute this safety profile to the horizontal design, limited tissue dissection, and hemostatic and lymphatic-sparing advantages conferred by tumescent infiltration.

In cases with minor wound dehiscence, conservative wound care was sufficient to achieve healing. For hypertrophic scars, management included silicone gel sheets and corticosteroid infiltrations, which prevented the need for surgical revision. Our results are consistent with the findings of Wollina et al. [15] and Bolletta et al. [31], who demonstrated successful outcomes in elderly or post-bariatric patients using TLA for body contouring procedures.

### 4.5. Economic and Logistical Considerations

From an economic and logistical standpoint, TLA offers multiple advantages [34,35]. Although the presence of a board-certified anesthesiologist remains essential to monitor for potential local anesthetic systemic toxicity (LAST), the elimination of general anesthesia reduces resource utilization. Intralipid was kept readily available throughout all procedures as a safety measure. The ability to perform surgery without airway management improves intraoperative efficiency and decreases room turnover times.

### 4.6. Study Limitations

The restricted use of horizontal-only resection limits the generalizability of our findings to patients with mild-to-moderate deformities. Moreover, as all patients included in this study presented with Grade 1 or 2 deformities on the Pittsburgh Rating Scale, the applicability of our findings to patients with more severe thigh laxity (Grades 3–4), particularly in post-bariatric populations, remains limited. These cases often require vertical or combined excisions and may present different complication profiles and technical challenges not addressed in the present analysis. Further comparative studies are needed to evaluate the efficacy of TLA in patients requiring vertical excisions or combined procedures. Additionally, longer follow-up may be warranted to assess scar quality, long-term tissue stability, and the risk of recurrence of ptosis [36,37].

### 4.7. Limitations

This study presents certain limitations related primarily to its design and scope. As a retrospective analysis without a comparative cohort undergoing medial thigh lift under spinal or general anesthesia, it does not allow for direct statistical evaluation of potential differences in outcomes between anesthetic approaches. Moreover, the patient population was relatively homogeneous, comprising individuals with moderate tissue laxity, which may limit the generalizability of our findings to patients with more severe post-weight-loss deformities. The absence of long-term follow-up data limits assessment of scar quality and tissue stability over time. Finally, while no systemic complications were observed, the sample size may not have captured rarer events. Nonetheless, the low complication rates and consistently positive outcomes found in this study support the reproducibility and clinical viability of tumescent anesthesia in appropriately selected candidates. Additionally, although our follow-up period extended to 12 months, longer-term assessment (18–24 months) would be necessary to fully evaluate scar maturation and the potential recurrence of ptosis, which may not yet be apparent at the one-year mark. Future prospective studies with extended surveillance are needed to confirm the duration of outcomes.

### 4.8. Clinical Implications

The findings of this study have implications for the practice of esthetic and post-bariatric body contouring surgery. The feasibility of medial thigh lift procedures under pure tumescent local anesthesia (TLA) without sedation demonstrates that effective, reproducible, and safe results can be achieved in a fully outpatient-based setting. This approach may be particularly beneficial for patients with contraindications to general anesthesia or those seeking reduced perioperative morbidity. The elimination of sedation and drains, combined with shortened operative times, may enable broader access to this procedure, especially in high-volume or resource-constrained environments. The minimal complication rate (23.2%) and absence of major systemic events reinforce the role of TLA as both an anesthetic modality and an element in optimizing perioperative safety and cost-effectiveness.

## 5. Conclusions

This study demonstrates that medial thigh lift performed under pure tumescent local anesthesia (TLA), without sedation or general anesthesia, is a safe and effective alternative for selected patients with mild-to-moderate skin laxity. The use of a horizontal-only excisional approach minimized complication rates while maintaining high patient satisfaction. Our experience confirms that TLA allows for reliable anesthesia, reduced perioperative morbidity, and expedited recovery, enabling the procedure to be carried out entirely in an outpatient setting. These results support broader consideration of TLA-based protocols in esthetic surgery, though further studies are warranted to validate these findings in more diverse and complex patient populations (Figure 3).

## Figures and Tables

**Table 1:** Patient Characteristics and Surgical Outcomes

Patient ID	Age	BMI	ASA Status	Surgical Type	Liposuction	Laxity Cause	Complications	Revision Needed	Intraop Pain Rating	12 Month Satisfaction	Smoke	Diabetes	PRS Grade
P01	45	28	I	Excision only	No	Post-weight loss	None	No	Excellent	Very satisfied	no	no	2
P02	36	24.5	I	Excision only	No	Post-weight loss	Hypertrophic scar	No	Good	Very satisfied	no	no	2
P03	43	27.5	I	Excision + liposuction	Yes	Post-weight loss	Dog ear	Yes	Excellent	Very satisfied	no	no	2
P04	51	29	I	Excision only	No	Age	None	No	Excellent	Very satisfied	no	no	2
P05	40	26	I	Excision + liposuction	Yes	Post-weight loss	Wound dehiscence	No	Good	Very satisfied	yes	yes	2
P06	38	25	I	Excision + liposuction	Yes	Post-weight loss	None	No	Excellent	Very satisfied	no	no	2
P07	29	26	I	Excision only	No	Post-weight loss	None	No	Excellent	Very satisfied	no	no	2
P08	33	25	I	Excision + liposuction	Yes	Post-weight loss	Scar migration	No	Excellent	Very satisfied	no	no	2
P09	58	33	II	Excision + liposuction	Yes	Age	None	No	Excellent	Very satisfied	no	no	2
P10	36	31	II	Excision only	No	Post-weight loss	Dog ear	Yes	Excellent	Satisfied	no	no	2
P11	42	27	I	Excision + liposuction	Yes	Post-weight loss	None	No	Excellent	Very satisfied	no	no	2
P12	35	29	I	Excision + liposuction	Yes	Post-weight loss	None	No	Excellent	Very satisfied	yes	no	2
P13	28	31	II	Excision + liposuction	Yes	Post-weight loss	None	No	Excellent	Very satisfied	no	no	2
P14	39	26	I	Excision only	No	Post-weight loss	None	No	Excellent	Very satisfied	no	no	3
P15	52	27	I	Excision + liposuction	Yes	Age	None	No	Excellent	Very satisfied	no	no	2
P16	36	28.5	I	Excision + liposuction	Yes	Post-weight loss	Wound dehiscence	No	Excellent	Very satisfied	no	no	2
P17	43	29	I	Excision + liposuction	Yes	Post-weight loss	None	No	Good	Very satisfied	no	no	2
P18	45	30.5	II	Excision + liposuction	Yes	Post-weight loss	None	No	Good	Very satisfied	no	no	2
P19	46	32	II	Excision + liposuction	Yes	Age	None	No	Excellent	Very satisfied	no	no	2
P20	37	28	I	Excision + liposuction	Yes	Post-weight loss	None	No	Good	Very satisfied	yes	no	1
P21	36	26.5	I	Excision + liposuction	Yes	Post-weight loss	Scar migration	No	Excellent	Very satisfied	no	no	2
P22	45	28	I	Excision + liposuction	Yes	Post-weight loss	Scar migration	No	Good	Satisfied	no	no	2
P23	50	31	II	Excision + liposuction	Yes	Age	None	No	Excellent	Very satisfied	no	no	2
P24	61	26	I	Excision only	No	Age	Wound dehiscence	No	Excellent	Very satisfied	no	no	2
P25	36	25	I	Excision only	No	Post-weight loss	None	No	Good	Very satisfied	yes	no	1
P26	55	25	I	Excision only	No	Age	None	No	Excellent	Very satisfied	no	no	1
P27	47	27.5	I	Excision + liposuction	Yes	Post-weight loss	Hypertrophic scar	No	Excellent	Very satisfied	yes	no	2
P28	33	24	I	Excision only	No	Post-weight loss	None	No	Excellent	Very satisfied	no	yes	2
P29	45	29	I	Excision + liposuction	Yes	Post-weight loss	Wound dehiscence	No	Excellent	Very satisfied	no	no	2
P30	39	28	I	Excision + liposuction	Yes	Post-weight loss	Dog ear	Yes	Good	Satisfied	no	no	2
P31	42	24.5	I	Excision only	No	Post-weight loss	None	No	Good	Very satisfied	no	no	2
P32	50	27	I	Excision + liposuction	Yes	Age	None	No	Excellent	Very satisfied	no	no	2
P33	46	26	I	Excision + liposuction	Yes	Post-weight loss	Dog ear	Yes	Excellent	Very satisfied	yes	no	2
P34	41	29.5	II	Excision + liposuction	Yes	Post-weight loss	None	No	Excellent	Very satisfied	no	no	2
P35	36	23	I	Excision only	No	Post-weight loss	None	No	Excellent	Very satisfied	no	no	1
P36	29	28	I	Excision + liposuction	Yes	Post-weight loss	None	No	Good	Very satisfied	no	no	2
P37	31	27	I	Excision + liposuction	Yes	Post-weight loss	Wound dehiscence	No	Good	Very satisfied	yes	no	2
P38	43	31	II	Excision + liposuction	Yes	Post-weight loss	None	No	Good	Very satisfied	no	no	2
P39	35	26	I	Excision + liposuction	Yes	Post-weight loss	None	No	Excellent	Very satisfied	no	no	1
P40	41	31	II	Excision + liposuction	Yes	Post-weight loss	Scar migration	No	Good	Very satisfied	no	no	2
P41	49	27	I	Excision + liposuction	Yes	Age	Wound dehiscence	No	Good	Very satisfied	yes	yes	2
P42	37	28	I	Excision + liposuction	Yes	Post-weight loss	None	No	Excellent	Very satisfied	no	no	2
P43	51	25	I	Excision only	No	Post-weight loss	None	No	Good	Very satisfied	no	no	1

## Data Availability

The data presented in this study are available in the article.

## Abbreviations

The following abbreviations are used in this manuscript:

TLA	Tumescent Local Anesthesia
GA	General Anesthesia
BMI	Body Mass Index
ASA	American Society of Anesthesiologists
POD	Postoperative Day
OR	Operative Room
ECG	Electrocardiogram
IV	Intravenous
VTE	Venous Thromboembolism
TXA	Tranexamic Acid
HRQoL	Health-Related Quality of Life
